# Efficacy and safety of nivolumab in Japanese patients with first recurrence of glioblastoma: an open-label, non-comparative study

**DOI:** 10.1007/s10147-021-02028-1

**Published:** 2021-09-29

**Authors:** Tomokazu Aoki, Naoki Kagawa, Kazuhiko Sugiyama, Toshihiko Wakabayashi, Yoshiki Arakawa, Shigeru Yamaguchi, Shota Tanaka, Eiichi Ishikawa, Yoshihiro Muragaki, Motoo Nagane, Mitsutoshi Nakada, Satoshi Suehiro, Nobuhiro Hata, Junichiro Kuroda, Yoshitaka Narita, Yukihiko Sonoda, Yasuo Iwadate, Manabu Natsumeda, Yoichi Nakazato, Hironobu Minami, Yuki Hirata, Shunsuke Hagihara, Ryo Nishikawa

**Affiliations:** 1 Department of Neurosurgery, National Hospital Organization Kyoto Medical Center, 1-1 Fukakusa Mukaihatacho, Fushimi Ward, Kyoto, 612-8555 Japan; 2 Department of Neurosurgery, Osaka University Graduate School of Medicine, Osaka, Japan; 3 Department of Clinical Oncology and Neuro-Oncology Program, Hiroshima University Hospital, Hiroshima, Japan; 4 Department of Neurosurgery, Nagoya University Hospital, Nagoya, Japan; 5 Department of Neurosurgery, Kyoto University Graduate School of Medicine, Kyoto, Japan; 6 Department of Neurosurgery, Hokkaido University Hospital, Hokkaido, Japan; 7 Department of Neurosurgery, The University of Tokyo Hospital, Tokyo, Japan; 8 Department of Neurosurgery, University of Tsukuba Hospital, Ibaraki, Japan; 9 Department of Neurosurgery, Tokyo Women’s Medical University Hospital, Tokyo, Japan; 10 Faculty of Medicine, Department of Neurosurgery, Kyorin University, Tokyo, Japan; 11 Department of Neurosurgery, Kanazawa University Hospital, Kanazawa, Japan; 12 Department of Neurosurgery, Ehime University Hospital, Ehime, Japan; 13 Department of Neurosurgery, Graduate School of Medical Sciences, Kyushu University, Fukuoka, Japan; 14 Department of Neurosurgery, Kumamoto University Hospital, Kumamoto, Japan; 15 Department of Neurosurgery and Neuro-Oncology, National Cancer Center Hospital, Tokyo, Japan; 16 Department of Neurosurgery, Yamagata University Hospital, Yamagata, Japan; 17 Department of Neurological Surgery, Chiba University Hospital, Chiba, Japan; 18 Department of Neurosurgery, Niigata University Medical and Dental Hospital, Niigata, Japan; 19 Hidaka Center for Pathologic Diagnosis and Research, Hidaka Hospital, Gunma, Japan; 20 Department Medical Oncology/Hematology, Kobe University, Kobe, Japan; 21 Oncology Early Clinical Development Planning, Ono Pharmaceutical Co., Ltd, Osaka, Japan; 22 Department of Statistical Analysis, Ono Pharmaceutical Co., Ltd, Osaka, Japan; 23 Department of Neuro-Oncology/Neurosurgery, Saitama Medical University International Medical Center, Saitama, Japan

**Keywords:** Bayesian approach, Bevacizumab, Clinical Trial, Phase II, Glioblastoma, Nivolumab, Programmed cell death

## Abstract

**Background:**

An open-label, non-comparative study assessed the efficacy and safety of nivolumab in Japanese patients with first recurrence glioblastoma.

**Methods:**

Patients with first recurrence of histologically confirmed World Health Organization Grade IV glioma, after treatment with temozolomide and radiotherapy, received nivolumab 3 mg/kg every 2 weeks until confirmed disease progression (Response Assessment in Neuro-Oncology criteria) or toxicity. Primary endpoint was 1-year overall survival rate assessed by Bayesian approach. The prespecified efficacy criterion was that the Bayesian posterior probability threshold for exceeding the 1-year overall survival of bevacizumab (34.5%) from the Japanese phase 2 study (JO22506) would be 93%.

**Results:**

Of the 50 enrolled patients, 44 (88.0%) had recurrent malignant glioma (glioblastoma, gliosarcoma), and of these, 26 (59.1%) had at least one measurable lesion at baseline. The Bayesian posterior mean 1-year overall survival (90% Bayesian credible intervals) with nivolumab was 54.4% (42.27–66.21), and the Bayesian posterior probability of exceeding the threshold of the 1-year overall survival rate of bevacizumab (34.5%) was 99.7%. Median (90% confidence interval) overall and progression-free survival was 13.1 (10.4–17.7) and 1.5 (1.4–1.5) months, respectively. One partial response was observed (objective response rate 1/26 evaluable patients [3.8%]). Treatment-related adverse event rates were 14.0% for Grade 3–4 and 2.0% for Grade 5; most adverse events resolved and were manageable.

**Conclusions:**

The 1-year overall survival with nivolumab monotherapy in Japanese patients with glioblastoma met the prespecified efficacy criterion. The safety profile of nivolumab was consistent with that observed in other tumor types.

**Clinical Trial Registration:**

JapicCTI-152967.

**Supplementary Information:**

The online version contains supplementary material available at 10.1007/s10147-021-02028-1.

## Introduction

In Japan, approximately 4000–5000 new cases of gliomas are reported each year [1, 2]. Despite treatment, patient outcomes remain poor in Japan [3], with a 5-year overall survival (OS) rate of 15.5%, a median OS of 18 months, and a local recurrence rate of 51% recorded [4]. Different treatment guidelines have been created for recurrent glioblastoma across the globe, but no standard treatment regimen has been established [5–7]. Currently, surgical re-excision or systemic and local chemotherapy with temozolomide, nitrosoureas, bevacizumab, or stereotactic irradiation to control localized lesions may be considered [7]. Bevacizumab was approved in Japan for recurrent glioblastoma based on a phase 2 trial (JO22506) of bevacizumab monotherapy in which the 6-month progression-free survival (PFS) rate was 33.9%, the 1-year OS rate was 34.5%, and the median OS was 10.5 months in Japanese patients with recurrent glioblastoma [8]. Although bevacizumab is often used for the treatment of recurrent glioblastoma, its clinical benefit is transient and variable [9, 10].

Recent advances in immuno-oncology provide evidence for the efficacy of programmed cell death-1 (PD-1)/programmed death-ligand 1 (PD-L1) blockade for a subset of cancers [11–15]. Several preclinical studies have demonstrated PD-L1 expression status in human glioma tissues [11, 16]; however, evidence of the efficacy of PD-1/PD-L1 antibody immunotherapy in glioblastoma is limited. Nivolumab, an anti-PD-1 monoclonal antibody [17], is approved in the United States, European Union, and Asia for the treatment of several cancer types [18]. Considering the link between PD-L1 expression and glioblastoma, nivolumab was hypothesized to be a potential therapeutic agent for the treatment of glioblastoma. Preliminary studies and case reports have shown benefits with nivolumab for glioblastoma; however, further research is needed [11]. The CheckMate 143 study (NCT02017717) evaluated the efficacy and safety of nivolumab vs bevacizumab in non-Japanese patients with recurrent glioblastoma [19]. Although the CheckMate 143 study (369 patients) did not demonstrate improved OS with nivolumab compared with bevacizumab (median OS, 9.8 vs 10 months), the median duration of response in evaluable patients was longer in the nivolumab group (11.1 months) than the bevacizumab group (5.3 months) [19]. These data suggest that nivolumab may offer some benefit to patients with recurrent glioblastoma. The objective of the current study (ONO-4538-19), run in parallel with the non-Japanese CheckMate 143 study, was to evaluate the efficacy and safety of nivolumab in Japanese patients with recurrent glioblastoma.

## Materials and methods

### Study design

This multicenter, open-label, non-comparative, non-randomized, phase 2 study evaluated the efficacy and safety of nivolumab in Japanese patients. The protocol was approved by the institutional review board of each study site (*n* = 20), was conducted between October 2015 and April 2019, and consisted of screening, treatment, and follow-up periods. The cut-off date for the data in this report is December 2017.

The study was conducted in accordance with the principles of the Declaration of Helsinki and Good Clinical Practice guidelines, as well as all local laws and regulations. The study was registered at the Japan Pharmaceutical Information Center (www.japic.org; JapicCTI-152967). All patients provided written informed consent.

### Patients

Adults (aged ≥ 20 years) with a first recurrence of histologically confirmed World Health Organization Grade IV malignant glioma (glioblastoma or gliosarcoma) confirmed by magnetic resonance imaging per Response Assessment in Neuro-Oncology (RANO) criteria or by histopathological evidence were enrolled. Patients had received first-line treatment with temozolomide plus radiotherapy (standard focally directed only), had a Karnofsky Performance Status (KPS) ≥ 70, and a life expectancy of ≥ 12 weeks. Patients with no measurable lesion or those with an interval of ≥ 28 days post–surgical resection after the first recurrence were also eligible.

Main exclusion criteria included patients with secondary glioblastoma (i.e., progress from low-grade diffuse astrocytoma, anaplastic astrocytoma, etc.) or extracranial metastatic or leptomeningeal disease, patients with multiple primary cancers, patients receiving treatments other than surgical therapy for recurrent glioblastoma, and patients with escalating or chronic supraphysiological doses of corticosteroids for disease control. Also excluded were patients receiving prior treatment with carmustine wafers (except when administered as first-line treatment and ≥ 180 days prior to randomization), bevacizumab, other monoclonal antibodies targeting vascular endothelial growth factor receptors, or antiangiogenic therapy, and patients with prior PD-1/PD-L1 or cytotoxic T-lymphocyte-associated protein targeted therapies.

### Treatment

Nivolumab 3 mg/kg was administered intravenously for approximately 60 min on day 1 of each 2-week cycle (same regimen as CheckMate 143) [19], with the first dose administered within 7 days of enrollment and at least a 10-day interval between doses. Treatment was continued until confirmed disease progression (by RANO criteria) or development of toxicity. However, continued treatment was permitted until re-confirmation of progression approximately 3 months after initially meeting RANO progressive disease (PD) criteria. There were no restrictions on using bevacizumab to treat worsening glioblastoma-associated symptoms post-second recurrence or disease progression confirmation.

### Endpoints and assessments

The primary efficacy endpoint was the 1-year survival rate, defined as the proportion of patients alive at 1 year since day 1 of treatment administration. Other endpoints included: best overall response per RANO criteria—the percentage of patients with complete response (CR) sustained for at least 4 weeks, partial response (PR) sustained for at least 4 weeks, stable disease (SD), PD, and non-evaluable disease; objective response rate (ORR), defined as the percentage of patients whose best overall response was a confirmed CR or PR (central and investigator assessment); OS; PFS (central and investigator assessment); and percentage change and maximum percentage change from baseline in sum of the products of maximal perpendicular diameters (SPD) of measurable lesions (investigator assessment).

Subgroup analyses of OS based on age, sex, KPS, *O*^*6*^-methylguanine-DNA methyltransferase (*MGMT*) methylation status, PD-L1 status, and corticosteroid use were conducted. Safety endpoints included the type, frequency, severity, and seriousness of adverse events (AEs) and the causal relationship with nivolumab. Types of AEs were assessed according to the Medical Dictionary for Regulatory Activities, version 20.1; AE grades were classified according to the Japanese translation of the National Cancer Institute Common Terminology Criteria for Adverse Events, version 4.0.

### Statistical methods

Efficacy was assessed using the full analysis set (FAS), which consisted of patients who received at least one dose of nivolumab, were compliant with Good Clinical Practice, and had Grade IV malignant glioma confirmed by central pathological review. Safety endpoints were assessed using the safety analysis set (SAS), which consisted of patients who received at least one dose of nivolumab.

A Bayesian approach [20–22] was used to assess the primary endpoint. At the time this study was planned, CheckMate 143 results were not available; therefore, the efficacy criterion of this study was based on the Japanese JO22506 study [8]. The prespecified efficacy criterion was that the posterior probability of the 1-year survival rate with nivolumab in this study exceeding the threshold 1-year survival rate with bevacizumab (34.5% [90% confidence interval (CI) 20.0–49.0]) from the JO22506 study would be more than 93%. Details of the probability density function are provided in Supplementary Material (Online Resource 1). To compute a 90% Bayesian credible interval, the quantiles of the posterior distribution used in this study were 0.05 and 0.95. Assuming the 1-year survival rate of nivolumab would be 49.0% based on the 1-year survival rate in the JO22506 study, and using the same assumed hazard ratio (HR) as the CheckMate 143 study (0.67) [19], the estimated sample size was 42 patients calculated using the Bayesian method [23]. However, to allow for sufficient patient numbers with confirmation of glioma diagnosis by central pathological review, a target sample size of 45 was selected. A uniform prior distribution of Beta (1,1) was selected for the prior distribution of the 1-year OS rate.

Best overall response and the ORR and their 90% CIs were calculated using the Clopper-Pearson method. Median OS and PFS with its 90% CIs, as well as OS and PFS rates at months 6, 12, 18, and 24, were calculated using the Kaplan–Meier method. The CIs of medians were calculated using the Brookmeyer–Crowley method [24], and the CIs of rates were derived based on Greenwood’s formula using double logarithmic transformation. HRs and 90% CIs were estimated using the Cox unstratified proportional hazards model. Percentage change and the maximum percentage change in the SPD of measurable lesions were plotted for each patient using spider plots and waterfall plots, respectively. No additional analyses were conducted for missing data, nor were adjustment analyses by covariates performed.

## Results

### Baseline characteristics

Of 50 patients enrolled (Table 1), 44 patients (88.0%) had recurrent malignant glioma (glioblastoma and gliosarcoma). A total of 37 (74.0%) patients had at least one measurable lesion at baseline, and corticosteroids were used by six (12.0%) patients at a dose < 4 mg/day. A total of 46 patients (92%) discontinued treatment owing to disease progression (78%), dosing delay lasting > 6 weeks (4%), investigator decision (4%), or other reasons (6%).

**Table 1:** Baseline and clinical characteristics of the total study population

	Nivolumab (*N* = 50)
Sex	
Male	34 (68.0)
Female	16 (32.0)
Age, years	
< 65	37 (74.0)
65– < 75	12 (24.0)
≥ 75	1 (2.0)
Karnofsky performance status	
100%	5 (10.0)
90%	18 (36.0)
80%	11 (22.0)
70%	16 (32.0)
*MGMT* gene promoter methylation	
Unmethylated	10 (20.0)
Methylated	12 (24.0)
Unknown	2 (4.0)
Not performed	26 (52.0)
Histopathological diagnosis (central review)	
Glioblastoma	43 (86.0)
Gliosarcoma	1 (2.0)
Others^a^	6 (12.0)
Time from initial diagnosis to recurrence, median (range), months	9.2 (2.0–61.9)
Corticosteroid use at baseline^b^	
No	44 (88.0)
Yes	6 (12.0)
< 4 mg/day	6 (12.0)
≥ 4 mg/day	0
Prior systemic therapy	
No	0
Yes	50 (100.0)
Temozolomide	50 (100.0)
Carmustine wafers	12 (24.0)
Others	5 (10.0)
Number of lesions (investigator review), median (range)	2 (0–4)
Patients with ≥ 1 measurable lesion	
No	13 (26.0)
Yes	37 (74.0)
Sum of products of maximum perpendicular diameters of measurable lesions^c^ (investigator review), median (range)	978.6 (110.0–3215.9)
PD-L1 status	
1% positive	18 (36.0)
1% negative	20 (40.0)
Not measured	12 (24.0)

Values are *n* (%), unless otherwise stated

^a^Other histopathological diagnoses (based on central review assesment) included: anaplastic oligoastrocytoma (*n* = 1), anaplastic astrocytoma (*n* = 1), a diagnosis compatible with anaplastic astrocytoma (*n* = 2), no evidence of tumor (*n* = 1), and slight infiltration of isocytrate dehydrogenase 1-mutated glioma cells (*n* = 1)

^b^Based on average corticosteroid use 5 days prior to start of dosing in dexamethasone equivalents

^c^Analyzed only patients with ≥ 1 measurable lesion

*MGMT* O^−6^ methylguanine-DNA methyltransferase, *PD-L1* programmed death-ligand 1

For patients included in the FAS (*N* = 44), 26 (59.1%) patients had at least one measurable lesion at baseline determined by central pathological review, and corticosteroid use was low with only four patients (9.1%) using corticosteroids at a dose < 4 mg/day.

### Efficacy

In the FAS, the posterior mean (90% Bayesian credible intervals) of the 1-year survival rate with nivolumab monotherapy was 54.4% (42.27–66.21), and the posterior probability of exceeding the prespecified threshold 1-year survival rate (i.e., 34.5%) was 99.7% (Table 2). Thus, the 1-year survival rate with nivolumab met the efficacy criterion prespecified for this study, and the primary endpoint was met.

**Table 2:** Primary endpoint: 1-year survival rate (FAS)

	Nivolumab (*n* = 44)^a^
Posterior mean of the 1-year survival rate, %	54.4
Posterior mode	54.5
Posterior variance	0.53
90% Bayesian credible intervals	42.27–66.21
Posterior probability that the result of the study exceeds the threshold 1-year survival rate,^b^ %	99.7

^a^Includes one patient who had gliosarcoma

^b^The 1-year survival rate of 34.5% in the JO22506 study (phase 2 study of single-agent bevacizumab in Japanese patients with recurrent glioblastoma) was selected as the threshold 1-year survival rate

*FAS* full analysis set

Best overall response with central review was PR (one patient; 2.3%) resulting in an ORR of 1/26 (3.8%) in patients with measurable lesions (Table 3). Median duration of response for the one patient with PR was 5.5 months, with a time to response of 2.8 months. Best overall response with investigator review was PR (two patients; 4.5%). SD was observed for 4.5% and 11.4% of patients with central and investigator reviews, respectively; no patient had a CR. There was good agreement between central and investigator reviews for the proportion of patients with PD (52.3% and 54.5%, respectively).

**Table 3:** Best overall response per RANO criteria (FAS)

Best overall response	Nivolumab (*n* = 44)	
	Central review	Investigator review
CR	0	0
PR	1 (2.3)	2 (4.5)
SD	2 (4.5)	5 (11.4)
PD	23 (52.3)	24 (54.5)
NE	18 (40.9)	13 (29.5)
No measurable lesion	16 (36.4)	11 (25.0)
Other reasons	2 (4.5)^a^	2 (4.5)

Values are *n* (%)

^a^Includes two patients who did not have a central radiologic review

*CR* complete response, *FAS* full analysis set, *NE* not estimable, *PD* progressive disease, *PR* partial response, *RANO* Radiologic Assessment in Neuro-Oncology criteria, *SD* stable disease

**Fig. 1:**
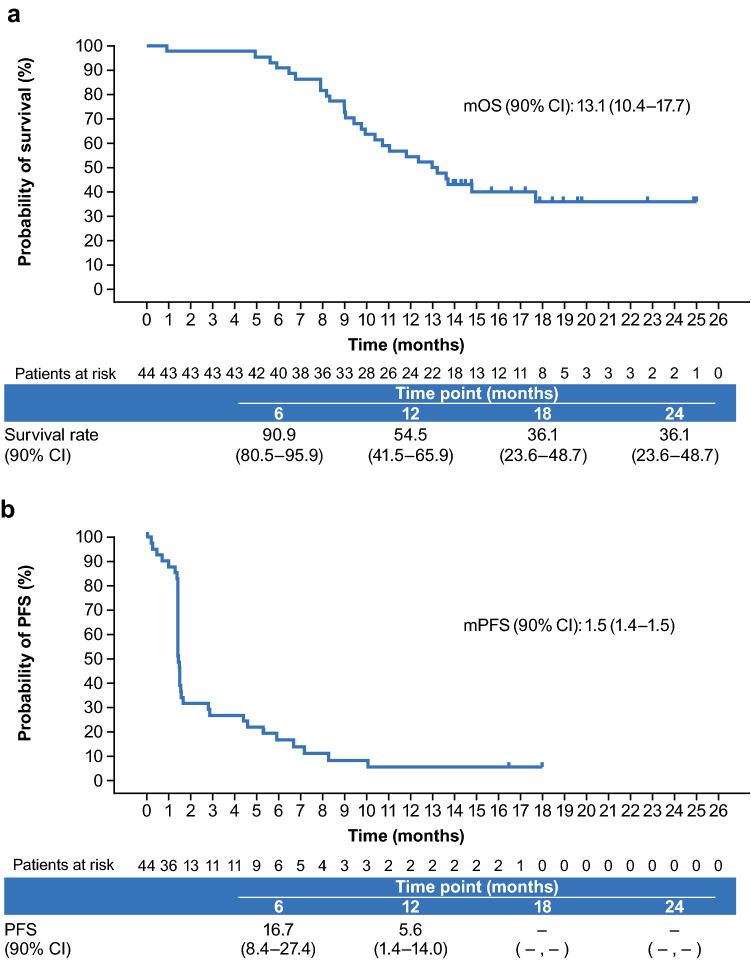
**a** Overall survival and **b** progression-free survival by central assessment. Vertical dashes represent censored observations. *CI* confidence interval, *mOS* modified overall survival, *mPFS* modified progression-free survival, *PFS* progression-free survival

**Fig. 2:**
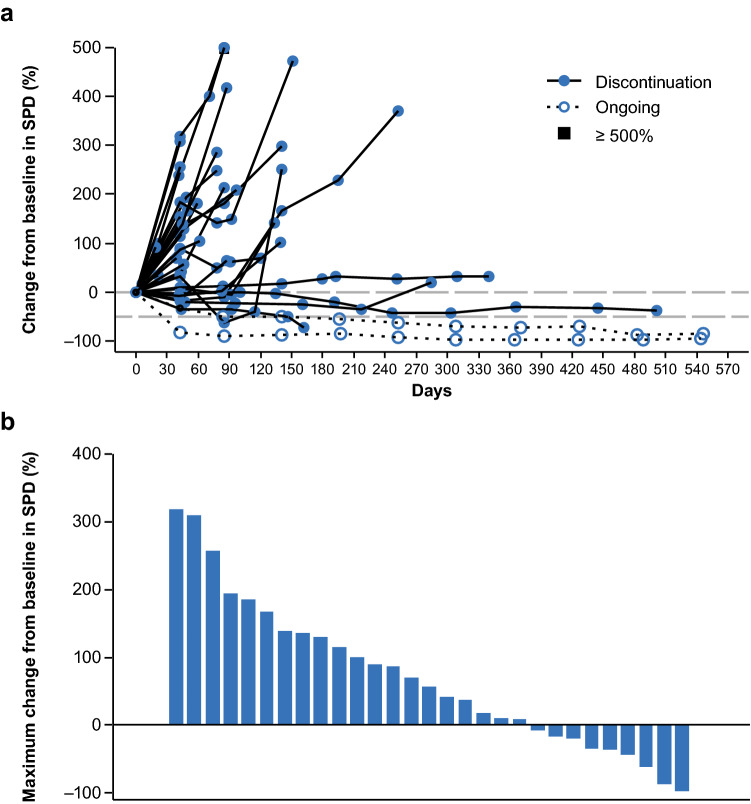
**a** Percentage change and **b** maximum percentage change from baseline in SPD of measurable lesions by investigator assessment. Panel **a** includes measurements from baseline to follow-up (including measurements after PD documentation). *N* = 31 patients; 13 patients who had a best overall response of NE^a^ were excluded. Panel **b** includes measurements from baseline up to PD documentation. *N* = 29; 13 patients who had a best overall response of NE^a^ and two patients with no MRI prior to a diagnosis of PD based on clinical deterioration were excluded. ^a^Where NE was owing to no measurable lesion available by investigator review or no evaluable MRI scans available after dosing. *MRI* magnetic resonance imaging, *NE* not estimable, *PD* progressive disease, *SPD* sum of the products of maximal perpendicular diameter

Median (90% CI) OS was 13.1 months (10.4–17.7) (Fig. 1a), and OS rates at 6, 12, 18, and 24 months were 90.9%, 54.5%, 36.1%, and 36.1%, respectively. Median (90% CI) PFS by central assessment was 1.5 months (1.4–1.5) (Fig. 1b). The size of the measurable lesion decreased in approximately 30% of patients with measurable lesions, and the antitumor effects were sustained in some of the patients with reduced measurable lesions (Fig. 2). Following nivolumab treatment, the switching rate to bevacizumab for the treatment of secondary recurrence was 65.9% (29/44 patients).

Subgroup analysis of OS was consistent with the primary analysis of OS (Table 4). Items that showed a measurable difference in median OS of more than 1 month between subgroups were KPS, *MGMT* promoter methylation, and PD-L1 status. There was a trend for longer median (90% CI) OS with increasing KPS score (KPS 100% or 90% vs 80% or 70%: HR, 0.55; 90% CI 0.29–1.05) and in patients with evidence of *MGMT* methylation (methylation vs unmethylation: HR 0.44; 90% CI 0.17–1.15), whereas, it was shorter for patients with PD-L1 positivity (1% cut-off) (PD-L1 positive vs PD-L1 negative: HR 3.03; 90% CI 1.44–6.36). For subgroups of female sex, age ≥ 65 years, and patients with baseline corticosteroid use, the median OS was not reached. In the majority of patients, specimens obtained at operation for newly diagnosed glioblastoma were used for PD-L1 immunohistochemistry analyses.

**Table 4:** Subgroup analyses for OS (FAS)

Subgroup	Nivolumab (*n* = 44)	
	*n*	mOS (90% CI), months
Sex		
Male	31	12.4 (9.8–13.7)
Female	13	– (8.3, –)
Age, years		
< 65	32	11.4 (9.0–13.6)
≥ 65	12	– (9.4, –)
Karnofsky Performance Status		
100%	5	17.7 (8.2, –)
90%	16	14.0 (9.8, –)
80%	11	12.4 (7.9–13.7)
70%	12	10.5 (6.8, –)
*MGMT* gene promoter methylation		
Unmethylated	7	9.0 (5.6–12.4)
Methylated	11	14.8 (9.0, –)
Unknown	2	10.0 (6.8–13.6)
Not performed	24	15.7 (10.7, –)
Corticosteroid use at baseline		
No	40	13.1 (9.9–17.7)
Yes	4	– (5.0, –)
1% PD-L1 status		
Positive	17	10.7 (9.0–13.0)
Negative	18	17.7 (13.6, –)
Not measured	9	8.2 (6.5, –)

– indicates endpoint “not reached”

*CI* confidence interval, *FAS* full analysis set, *MGMT* O^−6^ methylguanine-DNA methyltransferase, *mOS* median overall survival, *OS* overall survival, *PD-L1* programmed death-ligand 1

### Safety

In the SAS (*N* = 50), 90% of patients treated with nivolumab experienced AEs, with treatment-related adverse events (TRAEs) and serious adverse events related to nivolumab observed in 48.0% (Grades 3–4, 14.0%) and 12.0% (Grades 3–4, 10%) of patients, respectively (Table 5). The most common AEs (frequency ≥ 10%) were fever, headache, lymphocytopenia, constipation, nasopharyngitis, increased γ-glutamyl transferase, insomnia, and brain edema. Drug-related AEs leading to treatment discontinuation were observed in four patients (8.0%). The total Grade 3–5 TRAE rate was 16.0%. Most AEs resolved and were manageable. Grade 5 rhabdomyolysis was observed in one patient (2.0%) during cycle 2. The patient contracted influenza 9 days after receiving study drug, with persistent pyrexia, followed by the occurrence of acute kidney injury due to rhabdomyolysis; the patient developed a respiratory disorder owing to pulmonary congestion and died.

**Table 5:** Summary of adverse events (SAS)

Adverse events, *n* (%)	All grades (*n* = 50)	Grades 3–4 (*n* = 50)
*All causality*		
All AEs	45 (90.0)	24 (48.0)
SAE	16 (32.0)	11 (22.0)
AEs leading to discontinuation	7 (14.0)	4 (8.0)
*AEs occurring in ≥ 10% of patients*		
Pyrexia	10 (20.0)	0
Headache	9 (18.0)	0
Lymphocyte count decreased	9 (18.0)	5 (10.0)
Constipation	8 (16.0)	0
Nasopharyngitis	7 (14.0)	0
γ-Glutamyl transferase increased	5 (10.0)	0
Insomnia	5 (10.0)	0
Brain edema	5 (10.0)	1 (2.0)
*TRAEs*		
All TRAEs	24 (48.0)	7 (14.0)
Serious TRAEs	6 (12.0)	5 (10.0)
TRAEs leading to discontinuation	4 (8.0)	2 (4.0)
*TRAEs occurring in ≥ 2 patients*		
γ-Glutamyl transferase increased	4 (8.0)	0
Lymphocyte count decreased	3 (6.0)	1 (2.0)
Brain edema	2 (4.0)	1 (2.0)
Diarrhea	2 (4.0)	0
Pyrexia	2 (4.0)	0
Hypopituitarism	2 (4.0)	0
Rash maculo-papular	2 (4.0)	1 (2.0)

*AE* adverse event, *SAE* serious adverse event, *SAS* safety analysis set, *TRAE* treatment-related adverse event

## Discussion

The results of this prospective study indicate that nivolumab may have clinical activity with evidence of acceptable toxicity in Japanese patients with recurrent glioblastoma. The safety profiles of nivolumab in this study were consistent with that of previous studies of nivolumab in multiple tumor types [25–30]. No new safety signals were identified. Implementing the Bayesian approach, the results of this study suggest that nivolumab may be at least as effective as bevacizumab in the treatment of recurrent glioblastoma in Japanese patients. However, a direct comparison of the efficacy of nivolumab in this study with that of bevacizumab in the JO22506 study is difficult considering the different patient background factors of each study (discussed in detail below), and the fact that in this study more than 60% of patients switched to bevacizumab treatment upon disease progression.

In this study, the posterior mean (90% Bayesian credible intervals) 1-year survival rate with nivolumab was 54.4% (42.27–66.21), and the observed posterior probability of exceeding the 1-year survival rate of 34.5% for bevacizumab in the JO22506 study [8] was estimated to be 99.7%, exceeding the prespecified threshold of 93%. These results may support the probability that nivolumab might be more effective than bevacizumab in a Japanese population. It is important to note that the threshold 1-year survival rate (34.5%) selected for this study was based on the JO22506 study, which included patients with both first and second recurrence glioblastoma [8], whereas, only patients with first recurrence were included in this study. After the planning of the current study was completed, the 1-year survival rate with bevacizumab in the CheckMate 143 study became available, which was 42.0% [19].

In this phase 2 study, both the 1-year survival rate (54.5%) and median OS (13.1 months) were numerically higher than that observed with bevacizumab (42.0% and 10.0 months, respectively) and nivolumab (41.8% and 9.8 months, respectively) in the CheckMate 143 study [19]. In CheckMate 143, the response rate was lower (7.8% vs 23.1%), and median PFS was shorter (1.5 vs 3.5 months, respectively) with nivolumab compared with bevacizumab, and there was no survival benefit (HR 1.04; 95% CI 0.83–1.30). The observed differences between these two studies may be attributed to the multiple nivolumab injections or the switch to third-line therapies (65.9% switched to bevacizumab) from early nivolumab discontinuations in the current study, or to differences in baseline characteristics, such as corticosteroid use or *MGMT* methylation. The proportion of patients with corticosteroid use at baseline in this study was low (9.1%) compared with the proportion of patients in the nivolumab group in the CheckMate 143 study (39.7%). However, for patients with no corticosteroid use at baseline, the median OS with nivolumab was comparable in this study and in CheckMate 143 (13.1 vs 12.6 months, respectively), which was slightly higher than with bevacizumab in CheckMate 143 (11.8 months) [19].

Bevacizumab has established efficacy in treating recurrent glioblastomas [8, 31, 32]. In this study, patients responded well to nivolumab and achieved longer OS (13.1 months) than that reported for bevacizumab (10.5 months) in Japanese patients with recurrent glioblastoma (JO22506) [8]; however, a total of 65.9% of patients in this study switched to bevacizumab upon disease progression (median PFS 1.5 months), and this may have impacted the 1-year OS rate. PFS and ORR were not improved with nivolumab in this study compared with bevacizumab (Online Resource 2). In addition, the lower use of corticosteroids and higher proportion of younger patients at baseline in the current study, and inclusion of patients with a second recurrence in the JO22506 study, may have influenced the difference in survival results. Median OS with bevacizumab treatment has been shown to be slightly influenced by corticosteroid use [19]. Patients in the JO22506 study with a second recurrence likely required corticosteroids to maintain a KPS ≥ 70%; thus, they may have had a worse prognosis than the patients included in the current study.

The use of a Bayesian approach allowed appraisal of the results of parallel trials in glioblastoma, which may assist in future clinical decision-making. Using prespecified, data-driven, and scientifically based success criteria resulted in a well-designed study. In addition, the inclusion of subgroup analyses permitted the assessment of clinical and tumor characteristics to predict the clinical activity of nivolumab. This study showed consistency with CheckMate 143 with regard to potential benefits in selected subgroups, such as patients with no baseline corticosteroid use and those with *MGMT* methylation, in whom the median OS was longer with nivolumab than with bevacizumab. However, the success criterion was based on only one endpoint, and the effects of other endpoints were not considered. Moreover, the PFS with nivolumab was shown to be less than 2 months. Other limitations of the study included the lack of a direct comparator, the small sample size, and the lack of generalizability of the results to non-Japanese patients. Therefore, the results of this study should be verified in larger comparative trials.

In conclusion, this study demonstrated that nivolumab has acceptable toxicity with potential clinical activity, according to preset criteria, in Japanese patients with glioblastoma with first recurrence. However, PFS and ORR were not improved compared with the JO22506 study.

## Supplementary Information

Below is the link to the electronic supplementary material.

Supplementary file1 (PDF 214 KB)
