# Supplementary material for: Efficacy and safety of nivolumab in Japanese patients with first recurrence of glioblastoma: an open-label, non-comparative study
Source: Int J Clin Oncol. 2021 Sep 29;26(12):2205–15. doi: 10.1007/s10147-021-02028-1 (PMC8580927; doi:10.1007/s10147-021-02028-1)
Supplement: Supplementary file 1 — Supplementary file1 (PDF 214 KB) [file 10147_2021_2028_MOESM1_ESM.pdf]

Online Resources

International Journal of Clinical Oncology

Title: Efficacy and safety of nivolumab in Japanese patients with first recurrence of glioblastoma: an open-label, non-comparative study

Authors: Tomokazu Aoki, Naoki Kagawa, Kazuhiko Sugiyama, Toshihiko Wakabayashi, Yoshiki Arakawa, Shigeru Yamaguchi, Shota Tanaka, Eiichi Ishikawa, Yoshihiro Muragaki, Motoo Nagane, Mitsutoshi Nakada, Satoshi Suehiro, Nobuhiro Hata, Junichiro Kuroda, Yoshitaka Narita, Yukihiko Sonoda, Yasuo Iwadate, Manabu Natsumeda, Yoichi Nakazato, Hironobu Minami, Yuki Hirata, Shunsuke Hagihara, Ryo Nishikawa

Corresponding author: Tomokazu Aoki, Department of Neurosurgery, National Hospital Organization Kyoto Medical Center, 1-1 Fukakusa Mukaihatacho, Fushimi Ward, Kyoto, 612-8555, Japan; Email: totorolangdom@yahoo.co.jp

Online Resource 1 Probability density function

Using the expected 1-year survival rate (*p**) and threshold 1-year survival rate (p_0), the sample size was determined based on Pr(*p*> p_0 | *data*), the posterior probability that the 1-year survival rate estimated from the results of the study (*p*) exceeds p_0, and on Pr (*p*<*p** | *data*), the posterior probability that *p* does not exceed *p**

$$\Pr\left(p > p_0 \mid data\right) = \frac{1}{B(a+S, b+F)} \int_{p_0}^{1} p^{a+S-1}(1-p)^{b+F-1}\,dp$$

$$\Pr\left(p < p^* \mid data\right) = \frac{1}{B(a+S, b+F)} \int_{0}^{p^*} p^{a+S-1}(1-p)^{b+F-1}\,dp$$

- S: Number of patients alive at 1 year
- F : Number of deaths at 1 year
- $p - Beta(a,b)$ (Prior probability)

$$Beta(a,b) = \int_0^1 t^{a-1}(1-t)^{b-1}\,dt$$

Online Resource 2 Baseline characteristics and efficacy in the current study of nivolumab, the JO22506 [1] study of bevacizumab, and the CheckMate 143 study [2]

	**This study**	**JO22506 study**	**CheckMate 143 study**	
	**(Nivolumab) (*N* = 44)**	**(Bevacizumab) (*N* = 29)**	**Nivolumab (*N* = 184)**	**Bevacizumab (*N* = 185)**
Sex				
Male	31 (70.5)	14 (48.3)	116 (63.0)	119 (64.3)
Female	13 (29.5)	15 (51.7)	68 (37.0)	66 (35.7)
Age, years				
< 65	32 (72.7)	19 (65.5)	142 (77.2)	156 (84.3)
≥ 65	12 (27.3)	10 (34.5)	42 (22.8)	29 (15.7)
Karnofsky Performance Status				
90–100%	21 (47.7)	17 (58.6)	113 (61.4)	103 (55.7)
70–80%	23 (52.3)	12 (41.4)	69 (37.5)	81 (43.8)
Corticosteroid use at baseline				
Yes	4 (9.1)	10 (34.5)	73 (39.7)	79 (42.7)
No	40 (90.9)	19 (65.5)	111 (60.3)	106 (57.3)
Recurrence status				
First	100 (100.0)	17 (58.6)	NA	NA
Second	0	12 (41.4)	NA	NA
MGMT promoter methylation status				
Unmethylated	7 (15.9)	NA	59 (32.1)	67 (36.2)
Methylated	11 (25.0)	NA	43 (23.4)	42 (22.7)
Not reported/unknown	26 (59.1)	NA	82 (44.6)	76 (41.1)
1-year survival rate, %	54.5	34.5	41.8[a]	42.0[b]
mOS, months				
All	13.1	10.5	9.8[a]	10.0[b]
No corticosteroid use at baseline	13.1[c]	NA	12.6[d]	11.8[e]
mPFS,[f] months	1.5	3.3	1.5[g]	3.5[h]
ORR[f,i]	1 (3.8)[j]	8 (27.6)	12 (7.8)[k]	36 (23.1)[l]

Values are n (%), unless otherwise stated

[a]*N* = 154

[b]*N* = 147

[c]*N* = 40

[d]*N* = 111

[e]*N* = 106

[f]For mPFS and ORR, RANO criteria were used in the current study, whereas MacDonald criteria were used in the JO22506 study

[g]*N* = 171

[h]*N* = 146

[i]ORR was in patients with measurable lesion

[j]*N* = 26

[k]*N* = 153

[l]*N* = 156

MGMT O^6-methylguanine-DNA methyltransferase, *mOS* median overall survival, *mPFS* median progression-free survival, *NA* not available, *ORR* objective response rate, *RANO* Radiologic Assessment in Neuro-Oncology

References

1. Nagane M, Nishikawa R, Narita Y, et al (2012) Phase II study of single-agent bevacizumab in Japanese patients with recurrent malignant glioma. Jpn J Clin Oncol 42(10):887–895. https://doi.org/10.1093/jjco/hys121

2. Reardon DA, Brandes AA, Omuro A, et al (2020) Effect of nivolumab vs bevacizumab in patients with recurrent glioblastoma: the CheckMate 143 phase 3 randomized clinical trial. JAMA Oncol 6(7):1003–1010. https://doi.org/10.1001/jamaoncol.2020.1024
